# Inhibition of enterovirus 71 replication and viral 3C protease by quercetin

**DOI:** 10.1186/s12985-018-1023-6

**Published:** 2018-07-31

**Authors:** Chenguang Yao, Caili Xi, Kanghong Hu, Wa Gao, Xiaofeng Cai, Jinlan Qin, Shiyun Lv, Canghao Du, Yanhong Wei

**Affiliations:** 1 National “111” Center for Cellular Regulation and Molecular Pharmaceutics, Key Laboratory of Fermentation Engineering (Ministry of Education), Hubei Provincial Cooperative Innovation Center of Industrial Fermentation, Hubei Key Laboratory of Industrial Microbiology, Sino-German Biomedical Center, Hubei University of Technology, Wuhan, 430068 China; 2 Merck Stiftungsprofessur Molekulare BiotechnologieInstitut für Molekulare Biowissenschaften Goethe Universität Frankfurt, 60438 Frankfurt am Main, Germany

**Keywords:** Quercetin, Enterovirus 71, Anti-viral activity, 3C protease(3C^pro^)

## Abstract

**Background:**

Enterovirus 71 (EV71) is one of the major causative agents of hand, foot, and mouth disease (HFMD), which is sometimes associated with severe central nervous system disease in children. There is currently no specific medication for EV71 infection. Quercetin, one of the most widely distributed flavonoids in plants, has been demonstrated to inhibit various viral infections. However, investigation of the anti-EV71 mechanism has not been reported to date.

**Methods:**

The anti-EV71 activity of quercetin was evaluated by phenotype screening, determining the cytopathic effect (CPE) and EV71-induced cells apoptosis. The effects on EV71 replication were evaluated further by determining virus yield, viral RNA synthesis and protein expression, respectively. The mechanism of action against EV71 was determined from the effective stage and time-of-addition assays. The possible inhibitory functions of quercetin via viral 2Apro, 3C^pro^ or 3D^pol^ were tested. The interaction between EV71 3C^pro^ and quercetin was predicted and calculated by molecular docking.

**Results:**

Quercetin inhibited EV71-mediated cytopathogenic effects, reduced EV71 progeny yields, and prevented EV71-induced apoptosis with low cytotoxicity. Investigation of the underlying mechanism of action revealed that quercetin exhibited a preventive effect against EV71 infection and inhibited viral adsorption. Moreover, quercetin mediated its powerful therapeutic effects primarily by blocking the early post-attachment stage of viral infection. Further experiments demonstrated that quercetin potently inhibited the activity of the EV71 protease, 3C^pro^, blocking viral replication, but not the activity of the protease, 2A^pro^, or the RNA polymerase, 3D^pol^. Modeling of the molecular binding of the 3C^pro^-quercetin complex revealed that quercetin was predicted to insert into the substrate-binding pocket of EV71 3C^pro^, blocking substrate recognition and thereby inhibiting EV71 3C^pro^ activity.

**Conclusions:**

Quercetin can effectively prevent EV71-induced cell injury with low toxicity to host cells. Quercetin may act in more than one way to deter viral infection, exhibiting some preventive and a powerful therapeutic effect against EV71. Further, quercetin potently inhibits EV71 3C^pro^ activity, thereby blocking EV71 replication.

## Background

Enterovirus 71 (EV71) is a non-enveloped, positive-sense, single stranded RNA virus belonging to the family Picornaviridae, and a major pathogen causing hand, foot, and mouth disease in children under 5 years of age. EV71 was first isolated in California, USA [1]. Subsequently, EV71-associated hand-foot- mouse disease (HFMD) outbreaks occurred around the world [2–7]. Clinical manifestations of EV71 infection range from mild HFMD to severe encephalitis, pulmonary edema, and even death [8, 9]. In recent years, EV71 was reported to cause several large-scale outbreaks of severe complications in children involving the central nervous system, resulting in fatalities [10, 11]. EV71 caused 126 deaths in 2008 in China [12, 13] and the largest outbreak in China occurred in 2010, with an estimated 1.7 million infections, of which 27,000 caused severe neurological complications, resulting in 905 fatalities [14]. Fatal cases were also reported in Asia as recently as 2012 [13].

No approved direct-acting antiviral drug is available for EV71 infection to date [15]. Thus, the development of effective anti-EV71 agent is quite urgent and possesses important significance. At present, the prevention of EV71 epidemics mainly depends upon public surveillance. Ribavirin, type I interferon, and pleconaril are the common agents used to treat EV71 infections [16, 17]. In addition, some compounds, such as rupintrivir [18, 19], showed activity against EV71 in both cell lines and animal models; however, clinical applications are not yet available, therefore more efforts are needed to develop drugs to conquer EV71 infections.

The RNA genome of EV71 encodes a large precursor polypeptide that is processed by viral protease to generate viral structural (VP1, VP2, VP3, and VP4) and nonstructural (2A, 2B, 2C, 3A, 3B, 3C, and 3D) proteins [20]. Proteases play essential roles in the enteroviral life cycle. The virus polyprotein is co- and post-translationally processed by the viral proteases 2Apro, 3Cpro, and 3CDpro, the latter being the precursor of 3Cpro and the RNA-dependent RNA polymerase, 3Dpol. The first cleavage reaction in all enteroviral polyproteins is catalyzed by the 2Apro. These key viral proteases are the most prominent targets for consideration for development of antiviral agents [9, 21].

Quercetin, one of the most widely distributed flavonoids in plants, has been studied for many years because of its anti-inflammatory and anti-cancer properties [22], and has also been demonstrated to inhibit various viral infections. It has been reported to inhibit HIV reverse transcriptase and other retroviruses, such as herpes simplex virus type 1 (HSV-1), poliovirus type 1, respiratory syncytial virus (RSV) [23], and to significantly decrease the production of infectious particles by hepatitis C virus (HCV) and affect virion integrity [24]. Quercetin has also been reported to exhibit antiviral activity against HBV, influenza A virus (IAV) H1N1, and DENV-2 in vitro [25–27]. However, previous reports on the anti-EV71 activity of quercetin described only basic evaluation of its effects [28–30] and investigation of the anti-EV71 mechanism has not been reported to date.

Here, we studied the inhibitory activity of quercetin against EV71 and explored its anti-EV71 modes of action. We observed that quercetin showed powerful therapeutic effects, exhibited a degree of preventive activity against EV71, and also inhibited viral adsorption. Quercetin potently inhibited EV71 3C^pro^ activity, blocking EV71 replication. These findings are of potential relevance to the development of clinical therapeutics against EV71 infections.

## Methods

### Cells, viruses, and compounds

Human rhabdomyosarcoma cells (RD) and African green monkey kidney cells (Vero) were cultured in Dulbecco’s modified Eagle’s medium (DMEM, Gibco) supplemented with 10% fetal bovine serum (FBS) at 37 °C under a 5% CO_2_ atmosphere. EV71 (strain SK-EV006) with GFP (EV71-GFP) was kindly supplied by Prof. Bo Zhang from Wuhan Institute of Virology. Live EV71 strain wuhan/3018/2010virus was kindly provided by Prof. YingZhu (State Key Laboratory of Virology, College of Life Sciences, Wuhan University, China) and propagated in the RD cell line. Virus titer was determined by the standard TCID_50_ method [31]. Quercetin (C15H10O5; CAS no. 117–39-5; molecular weight [MW] 302.24) was purchased from Calbiochem (Germany) and prepared in DMSO. Ribavirin, used as a positive control, was purchased from Sigma Chemical Co. Stock solutions of drugs were prepared in DMSO (final concentration 0.1%). A fluorogenic peptide Dabcyl-RTATVQGPSLDFE-Edans, corresponding to the EV71 polyprotein autoprocessing site between 3B–3C, was purchased from Shanghai Kai Jing Biotechnology Co. Ltd.

### Antiviral activity and cytotoxicity

The anti-EV71 activity of quercetin was evaluated by phenotype screening. Briefly, RD cells were seeded in 96-well microtiter plates, after an overnight culture at 37 °C in 5% CO_2_, infected with 100 TCID_50_ of EV71-GFP virus (EV71 labeled with a stable eGFP reporter) [32] for 1.5 h, then incubated in a quercetin dilution series (3–100 μM) for 48 h, and the expression level of GFP monitored using an epifluorescence microscope.

The antiviral activity of quercetin against EV71 was further determined by a CPE reduction method. RD and Vero cells were seeded onto a 96-well culture plate. Next day, medium was removed and the cells were infected with 100 TCID_50_ of EV71 in the presence of serial dilutions of quercetin for 1.5 h at 37 °C, inocula were aspirated and the cells were further incubated with equal concentrations of quercetin at 37 °C in 5% CO_2_ for 2 days until appropriate CPE was achieved. Controls included uninfected cells and cells infected with virus in the absence of quercetin. The morphology of the cells to observation the effect of quercetin on EV71-induced CPE was observed under microscope, and images were recorded. The MTT assay was used to determine cell viability. The concentration required for the tested compound to reduce the CPE by 50% (EC50) was determined. The cytotoxicity of quercetin was also evaluated via the MTT-method after exposing uninfected cells to various concentrations of quercetin for 48 h at 37 °C. The results were expressed as CC50, defined as the concentration causing a 50% reduction or inhibition of cell viability. SIs were calculated as the ratio of CC50: EC50.

### Progeny virus titration

The titers of the virus stocks were determined by a TCID_50_ assay. Serially diluted viruses from 10^− 1^ to 10^− 8^ in DMEM were inoculated to RD cells in 96-well plates, and the cells were incubated for 2 days at 37 °C. TCID_50_ were calculated by counting the wells with cytopathic effect (CPE) in infected RD cells using the Reed–Muench method [31] .

### Modes of action assay

To explore the mechanisms underline the effects of quercetin on EV71 infection, three action modes (prevention action mode, mixture action mode, and treatment action mode) were applied. In the prevention action mode, RD cells were pre-incubated with various concentrations of quercetin for 2 h 37 °C, washed with PBS, and then infected with EV71 (100 TCID_50_). In the mixture action mode, an equal volume of EV71 suspension and quercetin, in DMEM were mixed and added to RD cells for 2 h at 37 °C, then inocula were aspirated and the cells were incubated with medium. In the treatment action mode, RD cells were first infected with EV71 (100 TCID_50_) for 2 h, the supernatants were removed and the infected cells were then treated with quercetin. The antiviral activities of quercetin were detected at 48 h post-infection using MTT assays.

### Time-of-addition assay

RD cells were infected with 100 TCID_50_ of EV71 and then 50 μM quercetin for different periods of time: -2 h to 12 h, 0–12 h, 2 h–12 h, 4 h–12 h, 6 h–12 h, and 8 h–12 h. At 12 h post infection, culture supernatants and cell lysates were collected following freeze-thaw cycles and then subjected to virus titration by TCID_50_ assay.

### RNA extraction and quantitative reverse transcriptase PCR

Total intracellular RNA was extracted using Trizol reagent (TaKaRa). Viral RNA samples were detected using an RT-PCR instrument (LightCycler 96, Roche) in accordance with the manufacturer’s instructions. A quantitative RT-PCR assay was performed using a PrimeScript RT reagent kit and SYBR Premix Ex Taq II (TaKaRa) following the manufacture’s protocol. Primer sequences were: EV71 VP1 forward, 5′-GCAGCCCAAAAGAACTTCAC-3′ and reverse, 5′-ATTTCAGCAG CTTGGAGTGC-3′; and β-actin, forward, 5′-GGCGGGACCACCATGTACCCT-3′ and reverse 5′-AGGGGCCGGACTCGTCATACT-3′. EV71 and β-actin transcript levels were determined using the ΔΔCT method.

### Immunofluorescence microscopy

RD cells (1 × 10^5^) were seeded in 24-well plates and incubated overnight. Cells were infected with 100 TCID_50_ of EV71 for 1.5 h with or without quercetin (50 μM) and the wells were fixed with 4% paraformaldehyde for 30 min at room temperature at various time points post-infection (p.i.). Plates were incubated with blocking solution (0.5% bovine serum albumin in phosphate-buffered saline (PBS)) for 1 h at room temperature and then reacted with mouse anti-enterovirus 71 monoclonal antibody diluted in blocking solution overnight at 4 °C. After plates had been washed three times with PBST (0.1% Tween-20 in PBS), they were incubated with the appropriate Alexa-Fluor-488-labeled secondary antibody and fluorescence evaluated using an OLYMPUS IX73 microscope.

### Flow cytometry

To assess levels of apoptosis, RD cells in 12-well plates were untreated or infected with 100 TCID_50_ of EV71. After viral adsorption, the cells were incubated in the absence or presence of 50 μM quercetin for 36–48 h, until the CPE of the virus control reached 70–90%. Cells were then stained with Annexin-V-fluorescein and propidium iodideand subsequently subjected to flow cytometry analysis, according to the manufacturer’s instructions. Apoptosis levels were determined using FlowJo software (USA).

### In vivo 2A protease activity assay

Plasmids encoding the 2A viral protease (pEGFP-C1-2A) or the 2A viral protease with a single point mutation (C110S) [pEGFP-C1-2Amut (C110S)] and a reporter plasmid, pCMV-FLuc, harboring firefly luciferase under the control of the CMV enhancer/promoter region, were co-transfected into RD cells seeded in 24-well plates using Lipofectamine 2000 transfection reagent (Life Technologies). Serial concentrations of quercetin were added 5 h post-transfection. After 24 h, cell lysates were collected and analyzed to determine FLuc activity using a Firefly luciferase assay system, following the manufacturer’s instructions (Multiskan®, Thermo).

### In vitro activity assay of 3C protease activity

#### Expression and purification of EV71 3C protease

The purification of EV71 3C protease was based on a previously reported protocol [33]. Briefly, the genomic region encoding EV71 3C protease was amplified and cloned into the pET-28a vector using the NcoI and HindIII restriction sites. Target proteins were expressed in *Escherichia coli* BL21 (DE3) after induction using isopropyl β-D-1-thiogalactopyranoside, and purified by affinity chromatography using a Ni-NTA column (Qiagen, Germany). The purified proteins were concentrated to 1 mg/mL in 20 mM Tris-HCl (pH 7.0), 500 mM NaCl, 2 mM DTT buffer for storage.

#### In vitro protease activity assay

EV71 3C^pro^ is a specific protease that recognizes peptide substrates containing a Q-G junction [34]. Thus, the substrate Dabcyl-RTATVQGPSLDFE- Edans was synthesized with fluorescence and quenching groups attached. Activity assays were performed using 1 μM 3C protease in 50 mM Tris-HCl, 200 mM NaCl, 2 mM DTT, and different concentrations of quercetin. Rutin was used as a positive control [35]. Reactions were incubated at room temperature for 6 h in a final volume of 100 μL. Subsequently, fluorogenic peptide substrate was added to a final concentration of 20 μM, then relative fluorescence was determined using an excitation wavelength of 340 nm and monitoring the emission at 500 nm every 30 s. All experiments were conducted in triplicate. Initial velocities of proteolysis were plotted as the function of quercetin concentrations by fitting the following equation: *A*(*I*) = *A*(0) × {1-([*I*])/([*I*] + IC_50_)}, where *A*(*I*) is the difference between the enzyme activity with quercetin concentration [*I*] and *A*(0), which is the enzyme activity without inhibitor.

### RNA elongation assay

The detailed protocol for the expression of EV71 3D polymerase and the polymerase elongation assay has been previously reported [36]. RNA elongation assays were performed using 0.1 μM 3D polymerase in 50 mM HEPES, 10 mM 2-mercaptoethanol, 5 mM MgCl_2_, 60 μM ZnCl_2_, 5 μM UTP, 0.4 μCi/μL [α-^32^P]UTP, 1.8 μM dT_15_-primer, 0.15 μM poly (rA) 300 and various concentrations of quercetin. Reactions were incubated for 5–10 min at 30 °C, and then quenched by adding 2 × gel loading buffer (90% formamide, 0.5% EDTA, 0.1% xylene cyanol and 0.1% bromophenol). After separating the reactions on 6 M urea polyacrylamide denaturing gels, RNA products were measured using a Cyclone Plus storage phosphor system (PerkinElmer).

### Molecular docking

The X-ray structure of EV71 3C protease (PDB no. 3SJO) was prepared using a Protein Preparation Wizard and Maestro 9.3 (Schrödinger, USA). The model of EV71 3C protease structure (EV71/wuhan/3018/2010) was used as a template to predicate whether quercetin affects 3C protease. The study of docking of EV71 3C protease with quercetin was performed using AUTODOCK 4.2.6, with atomic affinity potential energy calculated using AUTOGRID supporting software.

### Statistical analysis

The data shown are the means ± standard deviations (SD) of three independent experiments. The levels of significance for the differences between the test and control groups were analyzed using Student t-test, and the differences were considered significant if *P* values were < 0.05.

## Results

### Quercetin inhibits EV71 infection

**Fig. 1:**
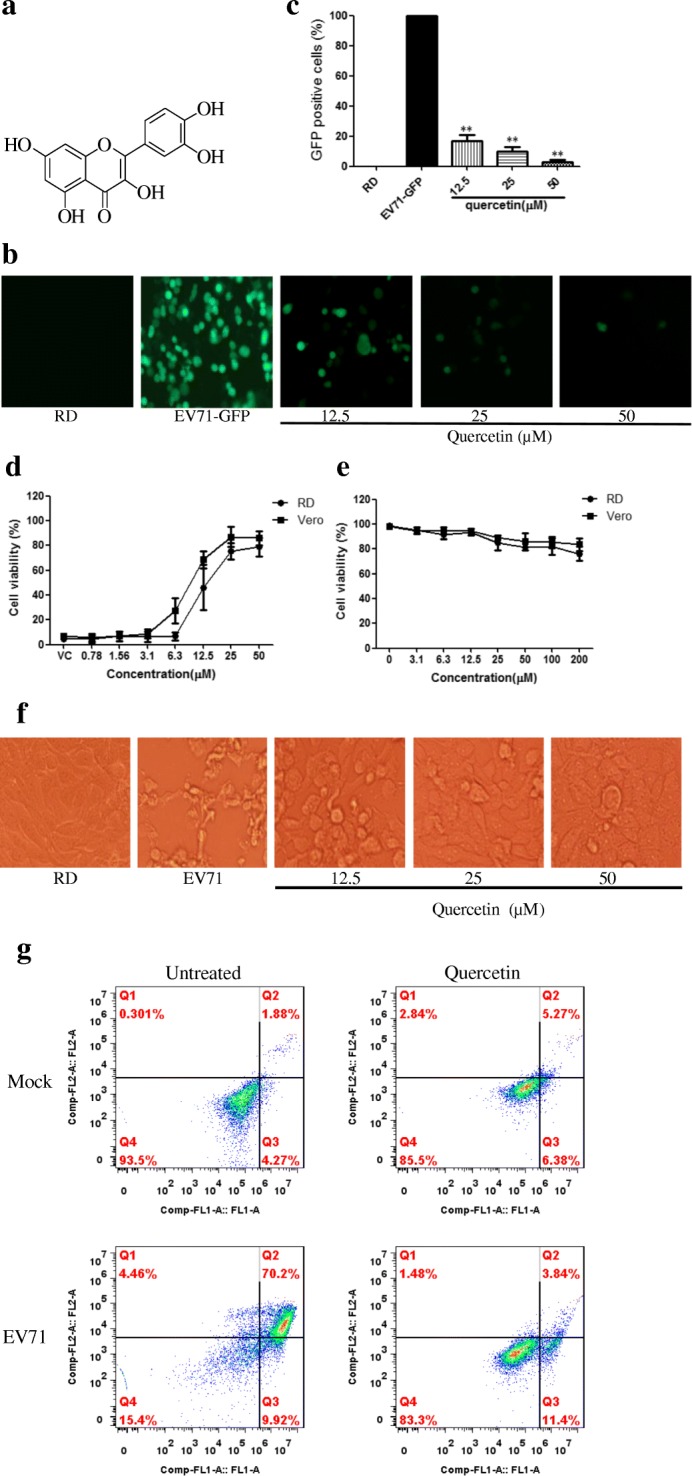
Quercetin inhibited EV71 proliferation. **a** The molecular structure of quercetin. **b** Reduction of EV71-GFP infection assay. RD cells were infected by EV71-GFP virus (100 TCID_50_), with or without treatments with various concentrations of quercetin. At 48 h pi, GFP expression was observed under a fluorescence microscope. **c** Antiviral activity was indicated by the reduction of the number of GFP-positive cells. **d** Antiviral activity of quercetin against EV71 in RD and Vero cells. Cells were infected with 100 TCID_50_ of EV71 mixed with serial dilutions of quercetin for 1.5 h, the inoculum was aspirated and cells were incubated with DMEM/quercetin for 48 h pi, the viability of the cells was determined with an MTT assay. VC, virus control. **e** The cytotoxicity of quercetin in RD and Vero cells. Cells were treated with serial concentrations of quercetin, the cell viability was determined by MTT assay after 48 h. **f** Morphology image of RD cells treated with quercetin (magnification, 20×). **g** The inhibitory effect of quercetin on EV71-induced apoptosis. RD cells were mock-infected or infected with EV71 (100 TCID_50_) in the presence or absence of quercetin (50 μM). The cells were stained with annexin-V-FITC/PI at 36–48 h pi., and cell apotosis and death was determined via a flow cytometry. The experiments were performed three times and the representative results were shown

The molecular structure of quercetin is presented in Fig. 1a. The anti-EV71 activity of quercetin was first investigated using a EV71-green fluorescent protein (GFP) virus phenotype screening assay. As shown in Fig. 1b and c, the number of GFP-positive cells reduced with increasing concentration of quercetin, suggesting that quercetin mediated concentration-dependent protection against EV71-GFP infection.

To further evaluate the anti-EV71 activity of quercetin, the inhibitory effects on the cytopathogenicity effect (CPE) induced by viral infection were examined in both RD and Vero cells by assessment of cell viability. The cytotoxic effects of quercetin were also evaluated. The results showed that quercetin exhibited low cytotoxicity in RD and Vero cells, with 50% cytotoxic concentration (CC50) values > 200 μM (Fig. 1e), and potently inhibited EV71 infection with a half-maximal effective concentration (EC50) of 12.1 μM for RD cells and 8.8 μM for Vero cells (Table 1). Compared with ribavirin (EC50 = 34.5 μM and 42.8 μM for RD and Vero cells, respectively) (Table 1), quercetin exhibited a more potent inhibitory effect against EV71. The dose-dependent antiviral effects are illustrated in Fig. 1d. Morphologically, EV71-infected RD cells showed a rounded appearance and detached from the dish in the absence of quercetin, whereas treatment with quercetin completely inhibited virus-induced CPE and no morphological cytotoxic effects were observed up to a concentration of 50 μM (Fig. 1f).

**Table 1:** Cytotoxicity and antiviral activity of quercetin against enterovirus 71 (EV71)

Cell types	Quercetin			Ribarivin^a^		
	EC50^b^ (μM)	CC50^c^ (μM)	SI^d^	EC50 (μM)	CC50 (μM)	SI
RD	12.1^e^±3.6	>200	>16.6	34.5±3.1	>200	>5.8
Vero	8.8±2.2	>200	>22.8	42.8±6.8	>200	>4.7

^a^Ribavirin, used as a positive control

^b^EC50: compound concentration required to achieve 50% protection from virus-induced cytopathogenicity

^c^CC50: compound concentration required to reduce cell viability by 50%

^d^SI (selectivity index): ratio by CC50/EC50

^e^Values represent the mean ± SD of three independent experiments

Previous studies have shown that EV71 induces apoptosis in infected cells [35]. We examined whether quercetin could attenuate EV71-induced apoptosis by analyzing RD cells infected with EV71 in the presence or absence of quercetin. Apoptosis levels were measured using an annexin-V-FITC/PI kit. As shown in Fig. 1g, EV71 infection contributed to apoptosis and cell death; RD cells infected with EV71 showed a significant fluorescence drift to the right (representative of early apoptosis) and the upper-right quadrant (representative of late apoptosis or death) compared with mock-infected cells. While similar fluorescence drift was barely detectable in the presence of 50 μM quercetin. These results indicate that quercetin could effectively inhibit virus-induced apoptosis and death of RD cells.

### Preliminary studies of the mechanism of action of quercetin against EV71

**Fig. 2:**
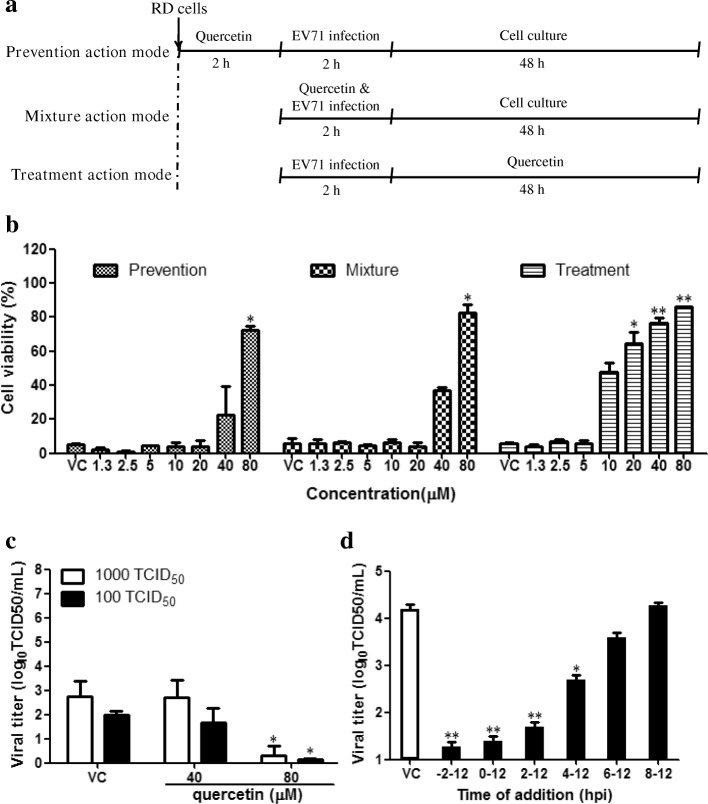
Analysis of effective stage. **a** The design of action mode of quercetin against EV71. **b** The antiviral effects were detected by measuring cell viability after 48 h of infection when cells were treated with quercetin before, simultaneously or after EV71 (100 TCID_50_) inoculation. **c** Analysis of the effect on EV71 adsorption. Mock- or quercetin-treated EV71 was inoculated onto RD cells and adsorbed for 2 h, the infected cells were harvested and then subjected to virus titrations using the TCID_50_ method. **d** Time-of-addition assay. 50 μM quercetin was added to RD cells at different time periods after EV71 infection. At 12 h pi, the progeny virus yield was determined by the TCID_50_ assay. (− 2–0 h: viral infection period; 0–12 h: the period for virus proliferation in the cells). VC, virus control

To identify the stage in the viral life cycle that was affected by quercetin, assays were performed using three different treatment protocols (Fig. 2a). As shown in Fig. 2b, quercetin-pretreated cells were resistant to subsequent infections with EV71 at a high concentration (80 μM) compared with the control group, indicating that quercetin was highly effective in influencing cellular function to prevent viral infection.

Similar results were obtained when quercetin was added to RD cells concurrently with EV71 (Fig. 2b), suggesting that quercetin can affect viral adsorption; however, the assay was cell-based and therefore necessitated cellular uptake of the test compound. Determination of cell viability 48 h after viral infection indicated that the inhibitory effect of quercetin on EV71 may result from the inhibition of viral replication in cells when the compound was also absorbed into cells, rather than only by inhibition of virus adsorption. Therefore, we performed an adsorption inhibition assay using intracellular virus titrations via the median tissue culture infective dose (TCID_50_) method. RD cells were infected with EV71 (100 and 1000 TCID_50_) and treated with 40 and 80 μM quercetin. After 2 h adsorption at 37 °C, the inoculum was discarded, the cells were washed and harvested following freeze-thaw cycles, and subjected to virus titration. Cells infected with EV71 in the absence of quercetin were used as the virus control. As illustrated in Fig. 2c, 80 μM quercetin decreased the virus titer significantly during virus attachment, with no apparent reduction when 40 μM quercetin was added. These results confirm that virus adsorption was quantitatively affected by quercetin.

Quercetin exhibited powerful therapeutic effects, with treatment at a concentration of 80 μM leading to cell viability of almost 100% (Fig. 2b), indicating that quercetin mainly blocked the post-attachment stage of viral infection.

To further understand the mechanisms of quercetin against EV71 propagation in cells, a time-of-addition experiment was performed. For this experiment, 50 μM quercetin was added to infected cultures at different time periods (with 2 h intervals) and the reductions in virus yield, relative to those of untreated cultures, were determined at 12 h p.i. As shown in Fig. 2d, when quercetin was present for the whole course of the replication cycle (− 2 to 12 h p.i.), the progeny virus titer was markedly reduced, and addition of quercetin during the 0–12 h and 2–12 h stages after viral infection (p.i) had a similar effect. Treatment during other periods following EV71 infection resulted in a gradual increase of viral yield, reflecting the reduction/loss of the antiviral effects of the compound. These data indicate that quercetin may exert its anti-EV71 effects through interference with the early events of viral replication.

### Quercetin strongly inhibited viral replication in RD cells

To further investigate the effect of quercetin on EV71 replication, the efficacy of quercetin in inhibiting viral progeny yield, viral RNA synthesis, and translation of viral protein were analyzed. Infected cells treated with or without 50 μM quercetin were harvested 4, 8, 16 and 32 h p.i. and progeny viral yields were determined using the Reed and Muench method. Quantitative reverse transcription polymerase chain reaction (qRT-PCR) and indirect immunofluorescence analysis of harvested cells were also carried out to determine the relative amounts of viral RNA and viral protein, respectively.

**Fig. 3:**
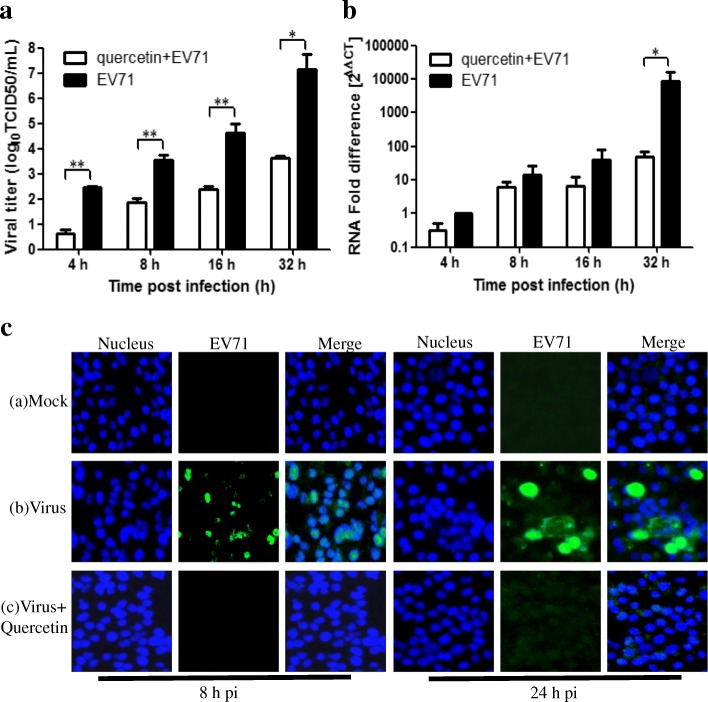
The effect of quercetin on EV71 replication in RD cells. RD cells infected with 100 TCID_50_ of EV71 were incubated in the absence or presence of 50 μM quercetin and harvested at the indicated times pi. **a** The progeny viral yields were determined. **b** Total RNA was extracted from cells and culture supernatants and EV71 RNA levels were measured. Cellular actin amplification was used for normalization. **c** EV71-protein was determined by indirect immunofluorescence using a mouse anti-enterovirus 71 monoclonal antibody and an Alexa-Fluor-488-conjugated AffiniPure goat anti-mouse IgG (H + L). The nucleus was stained with DAPI, the green foci indicate the presence of EV71 protein. Values represent the means ± SDs of three independent experiments. *P < 0.05; **P < 0.01, compared with EV71 control group

As shown in Fig. 3a, the virus titer continued to increase from 4 to 32 h in the virus control cells, indicating active viral replication in the cells following viral inoculation. Notably, less change was observed in cells treated with quercetin, and the inhibitory effect was most prominent at 32 h p.i, with an approximate 3.5 log reduction in viral replication. Fig. 3b illustrates the efficacy of quercetin in inhibiting viral RNA synthesis. Viral RNA became detectable in the virus control group during the first 4 h and was followed by significant increases at subsequent time points. Viral copy numbers in quercetin-treated RD cells were significantly lower than those in virus control cells, with the most prominent inhibition observed at 32 h. These results imply that quercetin targets viral replication in RD cells.

The influence of quercetin on EV71 replication at the level of translation was also determined. Viral protein immunofluorescence foci were absent in mock-infected control cells (Fig. 3c-a), demonstrating that the antibody was specific for EV71. The green immunofluorescence foci in the virus control group (Fig. 3c-b) were significantly more abundant than those in quercetin-treated cells (Fig. 3c-c), indicating that viral protein synthesis was suppressed by quercetin. These results led us to hypothesize that quercetin suppresses EV71 replication by inhibiting viral RNA and protein synthesis.

### Quercetin did not affect 2A^pro^ or 3D^pol^ activity

**Fig. 4:**
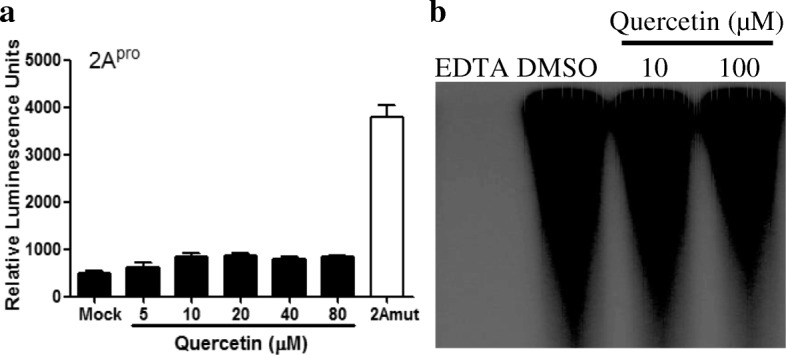
Effects of quercetin on EV71 2A^pro^ and 3D^pol^ activity. **a** Effect of quercetin on 2A^pro^ activity of EV71. RD cells were transfected with pEGFP-C1-2A or pEGFP-C1-2Amut (C110S) and reporter plasmid pCMV-FLuc, and then quercetin was added or not. After 24 h, the firefly luciferase activities were analysed. The data of three independent experiments are presented as the mean ± the standard deviation. **b** Effect of quercetin on EV71 3D RdRp primer extension assay. RNA polymerase assays contained poly (rA) 300, EV71 3Dpol, UTP, and [α-32P] UTP with or without quercetin (10 and 100 μM), the radioactivity was measured. The experiments were performed three times and the representative result was shown

Enterovirus 2A^pro^ cleaves viral polyproteins and cellular factors, and has multiple roles in regulation of various viral and cellular processes, including viral replication and cytopathogenesis [37, 38], therefore, the possible inhibitory function of quercetin via viral 2Apro was tested. RD cells were co-transfected with the reporter plasmid, pCMV-FLuc, harboring firefly luciferase, and the plasmid pEGFP-2A, or pEGFP-2Amut (C110S) (2Amut), as a negative control. After 24 h, firefly luciferase (FLuc) activities were analyzed. 2Apro can cleave the host cell translation protein, eIF4G [37], contributing to the shut-down of cellular cap-dependent translation. If 2A^pro^ activity was inhibited, firefly luciferase would be highly expressed, luminescence intensity increased, and vice versa. As shown in Fig. 4a, there was a significant reduction in the Renilla luciferase signal in cell lysates expressing 2A^pro^ compared with those expressing inactive 2A^pro^. When cells were treated with different concentrations of quercetin, there was no restoration of the reporter signal in RD cells co-transfected with the reporter plasmid and the 2A expression plasmid, indicating that quercetin did not inhibit the activity of EV71 2A^pro^.

EV71 3D^pol^ is an RNA-dependent RNA polymerase (RdRp) which is key in the process of viral RNA replication. We investigated the effect of quercetin on EV71 3D^pol^ activity in vitro by detecting the amount of radio-labeled UMP incorporated into poly (A) RNA in the presence of poly (A) templates and oligo (dT) primers. The rate of UMP incorporation by EV71 3D^pol^ in the presence of 0.5% dimethyl sulfoxide (DMSO; the vehicle used for dissolving quercetin) and EDTA were employed as negative and positive controls, respectively. The results presented in Fig. 4b demonstrate that UMP incorporation into RdRp reaction products was entirely inhibited in the presence of EDTA, whereas EV71 3D^pol^exhibited potent activity in the presence of 0.5% DMSO, and almost the same activity in the presence of 10 μM quercetin. Minimal inhibition was also observed by addition of 100 μM quercetin to the RdRp reaction. The results of this experiment indicate that quercetin did not interfere with EV71 3D^pol^ activity.

### Quercetin targets EV71 3C^pro^

**Fig. 5:**
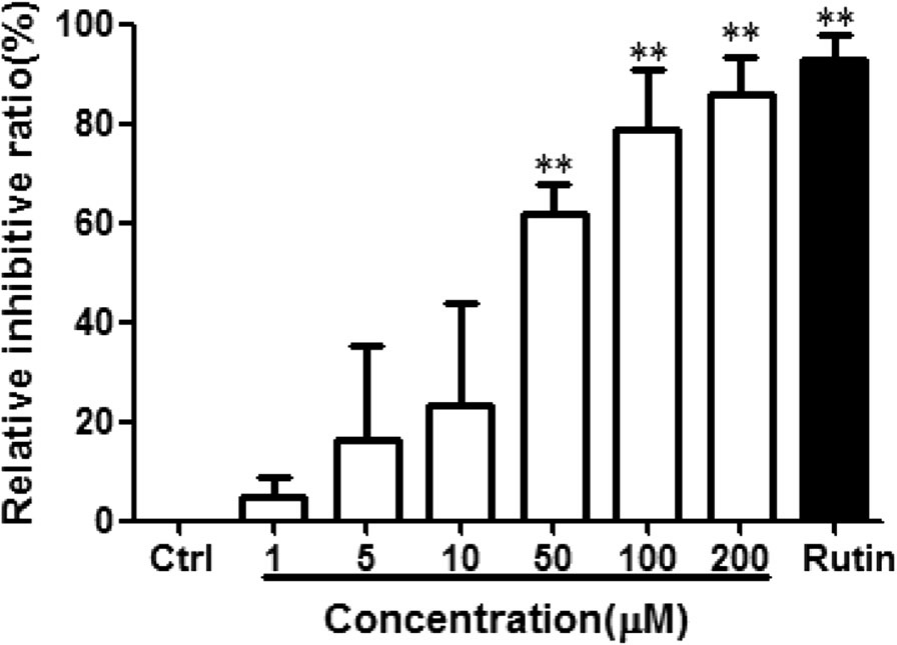
The inhibitory effect of quercetin on EV71 3C^pro^ activity. The synthetic peptide substrate containing Gln-Gly pairs corresponding to the cleavage site by 3C^pro^ was designed as substrate for EV71 3C^pro^. Various concentrations of quercetin were incubated with 3C protease and the fluorogenic peptides substrate at 37 °C. The data of three independent experiments are presented as the mean ± the standard deviation. **P < 0.01, compared with 3C^pro^control

EV71 3C^pro^ is vital for the maturation of virion particles, and an ideal drug target. To further identify the potential antiviral target of quercetin, its effects on 3C^pro^ activity were investigated. The peptide Dabcyl-KTSAVLQSGFRKME-Edans (incorporating the fluorescence quenching pair, Dabcyl–Edans) was chosen for use in an EV71 3C^pro^ assay, and the increase in fluorescence (indicating real-time peptide bond cleavage) monitored. Rutin, a well-known 3C^pro^ inhibitor, was used as a positive control. As illustrated in Fig. 5, quercetin significantly inhibited 3C^pro^ in a dose-dependent manner in vitro, and the EC50 of quercetin against 3C^pro^ was 30.3 μM. The presence of 200 μM quercetin reduced 3C^pro^ activity by 86%, comparable to the effects of rutin. These observations suggest that quercetin is a potent inhibitor of 3C^pro^.

### Interaction between quercetin and 3C^pro^

**Fig. 6:**
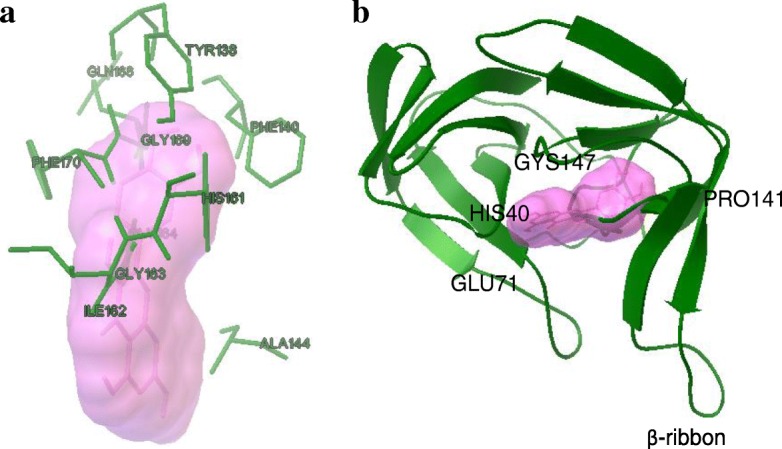
Molecular docking for the interaction between quercetin and EV71 3C^pro^. **a** Stick conformer diagram. **b** Molecular binding model for quercetin with viral 3C^pro^. The structure of EV71 3C^pro^ (PDB no. 3OSY) was used as a template. The interaction between quercetin and viral 3C^pro^ was detected using AUTODOCK 4.2. The color of O atom of quercetin is red

The crystal structure of EV71 3Cpro has been determined and reported previously [39]. A flexible surface loop β-ribbon is important for recognition of the P2–P4 region of the peptide substrate. The stabilization of the open β-ribbon conformation requires intermolecular hydrogen bonds and hydrophobic interactions, formed by Gly1-Gln168, Tyr122-Phe113, Gln121-Ser111, and a hydrophobic patch generated by interaction between Leu25 and Pro131, and Met109, Met112, Val114, and Phe140 [34]. To predict the interaction between EV71 3C^pro^ and quercetin, we tested the possible interaction and calculated the binding energy by molecular docking. We initially identified putative sites through which quercetin might bind to 3C^pro^ (Protein Data Bank [PDB] no. 3OSY) using this method. The binding free energy was calculated (Table 2) and the optimal value, indicating a site most likely to mediate interaction of quercetin with 3C^pro^, was chosen (Fig. 6). The results illustrate that quercetin is predicted to bind stably in the substrate recognition region (P2–P4) mainly through hydrogen bonds formed by residue Gly164 and hydrophobic interactions formed by residues Try138, Phe140, Ala144, His161, Ile162, Gly163, Gln168, Gly169 and Phe170 in 3C^pro^ (Fig. 6a). The molecular structure of quercetin bound to 3C^pro^ indicates that it partially occupies a catalytically important amino acid loop (aa) 140–147 (Fig. 6b).

**Table 2:** Docking conformation for quercetin with the viral 3C^pro^

Rank	Binding energy (kJ/mol)	Residue formed hydrogen bonds	Interaction of residue between 3C^pro^ and quercetin
0	-	Gly164	Tyr138、Phe140、Ala144、His161、Ile162、Gly163、Gly164、Gln168、Gly169、Phe170
1	-5.27	Pro5、Ser111	Met0、Arg4、Pro5、Ser111、Met112、Phe113
2	-5.03	Ser111	Arg4、Leu4、Pro5、Thr101、Ser111、Phe113、Pro115
3	-4.8	Val63、Ser177、Ala180、Ser181	Val60、Val63、Asp64、Leu74、Ser177、Ala180、Ser181
4	-4.31	Ser95、Thr96	Pro91、Glu92、Ser95、Thr96、Lys156、Arg176
5	-4.04	Arg39、Asn69、His133、Arg134	Arg39、Asn69、Leu70、Glu71、His133、Arg134
6	-3.86	Ala180	Asp58、Val60、Leu62、Leu74、Thr76、Ser177、Tyr178、Ala180、Ser181
7	-3.79	Thr135	His40、Glu71、Thr135、Gly163、Gly164、Phe170
8	-3.57	Gln22、His24、Phe25	Gln22、Gly23、His24、Phe25、His40、Cys147
9	-3.47	Asp64、Ser177	Val63、Asp64、Glu65、Ser177、Tyr178、Ala180、Ser181
10	-3.4	Lys130	Asp64、Glu65、Lys130、His133、Arg134、Lys175、Tyr178

Based on these results, we predicted that quercetin would be able to bind to P2–P4 and inhibit the interaction between the peptide substrate and the 3C^pro^ active site, thereby contributing to 3C^pro^ inactivation. These results indicate that quercetin may block substrate recognition to inhibit EV71 3C^pro^ activity.

## Discussion

HFMD is a common pediatric illness primarily caused by infection with EV71 [39, 40]. No effective or specific therapeutic measures against HFMD are available. Hence, an effective antiviral therapy against EV71 infection is urgently needed. Flavonoids are associated with multiple biological and pharmacological activities. Some flavonoids and isoflavonoids have also been reported to exhibit anti-EV71 activities [23, 26]; however, the majority of compounds were only evaluated as anti-EV71 agents and research into the anti-EV71 mechanism of flavonoids and isoflavonoids has rarely been reported. In this study, we tested the anti-EV71 activity of the natural flavonoid, quercetin, and explored its mode of action against EV71 for the first time.

In this study, a two-step detection system, consisting of an enhanced GFP reporter infection assay and a cell viability-based assay, was developed to assess the antiviral activity of quercetin against EV71. A major limitation of these assays is their inability to discriminate between drug toxicity and viral-induced CPE. Moreover, reporter virus-based assays are only suitable for determining the antiviral activity of non-toxic compounds, since toxic compounds decrease the fluorescence or luminescence signal. Thus, the EV71 antiviral assay used in this study was conducted by combining these two approaches. Moreover, EC50 values of quercetin against EV71 were also obtained by other approaches, including CPE microscopy and classic virus yield reduction assay (data not shown), in addition to the two assays described above. GFP reporter infection assays, using a full-length infectious clone, allowed screening for inhibitors of any step(s) of the replication cycle of EV71. CPE detection, MTT and virus yield assays reflected changes in cellular phenotypes, metabolism, and viral multiplication, respectively. The similarity of the EC50s obtained using these different approaches suggested that quercetin alone did not inhibit cell growth and could effectively inhibit viral infection.

Furthermore, we determined the CC50 values at a maximum quercetin concentration of 200 μM, because when the concentration was higher than 200 μM, a large amount of particles would adsorb on the cell surface, resulting in an inability to visualize the state of the cells, or to accurately determine cell viability using the MTT method. The value of CC50 for quercetin was > 200 μM, which was the same as the positive control, ribavirin. Quercetin exhibited dose-dependent antiviral effects against EV71, which meant quercetin could inhibit EV71 infection only when it reached a certain concentration. Actually, quercetin had an EC50 of 12.1 μM for RD cells and 8.8 μM for Vero cells against EV71 infection, with selective indexes of > 16.6 and > 22.8, respectively. An approximately ten-fold inhibitory activity against EV71 relative to ribavirin was observed. Selectivity index (SI) values ≥4 are considered suitable for antiviral agents [41], which suggesting that quercetin is an effective anti-EV71 agent with a relatively safe profile. Previous reports also demonstrated that quercetin showed potent anti-EV71 activity without toxic effects [29]. These results suggest that quercetin has potential for use in therapeutic application against EV71 infection.

An internal ribosomal entry site (IRES) required for viral protein translation is a potential EV71 drug target. As previously reported, several flavonoids, including kaempferol and hesperetin, exhibit antiviral activity against EV71 through inhibition of the IRES; however, quercetin does not inhibit IRES-driven translation [29]. Another dietary flavonoid, apigenin, suppresses EV71 replication through a novel mechanism by targeting trans-acting factors [28]. The results of this study demonstrated that quercetin was able to effectively inhibit EV71 replication by inhibition of the activity of the virus-encoded 3C^pro^, and the results verified that flavonoids with similar structures exert different mechanisms against EV71. Further study of the affinity of quercetin for 3C^pro^ is warranted to ascertain whether the compound affects other viral or cellular functions crucial for EV71 replication.

In this study, recombinant 3C^pro^ and fluorogenic substrate were used to evaluate the effect of quercetin on protease activity. The amounts of quercetin required to inhibit purified EV71 3C^pro^ activity in vitro were higher than those required to block EV71 replication, this is because the higher concentrations of 3C^pro^ present in vitro required more quercetin for inhibition.

Viral 3C^pro^ is a multifunctional protein involved in binding viral RNA and RNA replication, in addition to a number of other biological processes. It has also been reported that 3C^pro^ can impair host RNA processing and polyadenylation by cleavage of the cellular CstF-64 protein, leading to enhanced viral RNA replication [7]. The 3C protein can also trigger apoptosis through the caspase pathway in neuronal cells [42]. In this study, treatment of infected cells with quercetin reduced yields of viral RNA and inhibited EV71-induced apoptosis. These results led us to conclude that the inhibition of viral RNA synthesis and EV71-induced apoptosis in RD cells by quercetin was the result of its inhibition of viral 3C^pro^ activity.

The 3C-like protease (3CL^pro^) of severe acute respiratory syndrome-associated coronavirus (SARS-CoV) is vital for SARS-CoV replication, and quercetin displays strong inhibition of 3CLpro [43]. Quercetin has also been identified as a potent inhibitor of reverse transcriptases from rauscher murine leukemia virus (RLV) and human immunodeficiency virus (HIV) [44]. In this study, quercetin significantly inhibited the activity of 3Cpro of EV71. Further, we predicted the interaction between EV71 3C^pro^ and quercetin and calculated the binding energy using molecular docking. Docking calculations between 3C^pro^ and other flavonoids have been reported previously, and the accurate prediction of biomolecular conformation and binding energies of protein-ligand complexes exhibited similarities, including common binding sites Leu4, Leu8, Ser111, Met112, Phe113, and Pro115 [45, 46]. The amino acid sites predicted to be involved in 3Cpro-quercetin complexes included Met0, Arg4, Pro5, Ser111, Met112, Phe113, Leu4, Thr101, Pro115 (rank 1 and rank 2 in Table 2). Although these amino acid sites were explored for important roles in the interaction between EV71 3C^pro^ and other flavonoids (or its phosphate ester), they have not been identified as involved in biological catalysis to date. Analysis of the 3C^pro^-quercetin interaction revealed that quercetin interacted with the 3C^pro^ catalytic residues, His40, Glu71 and Cys147 (rank 7 and rank 8 in Table 2), which is different to other flavonoids, this binding may decrease EV71 3C^pro^ activity. Moreover, for the initial manner of binding, predicted by the docking results (rank 0 in Table 2), quercetin was predicted to interact with numerous residues in 3C^pro^, including Tyr138, Phe140, Ala144, His161, Ile162, Gly163, Gly164, Gln168, Gly169, and Phe170. The molecular binding model of the 3C^pro^-quercetin complex revealed that quercetin may stably insert into the substrate-binding pocket of EV71 3C^pro^ via numerous hydrogen bond interactions. Similar binding mechanisms have been reported for the interactions between flavonoids and 3CLpro in SARS-CoV [43], but not EV71. Therefore, these modes of interaction between different flavonoids and various viruses could be used as a reference for the design of 3C^pro^ inhibitors.

EV71 infection is often accompanied by the apoptosis of host cells, and it can be a mechanism for viral spread to neighboring cells, which may promote culminating a lytic infection and cause viremia and severe central nervous system complications. Here, quercetin was found to protect host cells from apoptosis induced by viral infections, suggesting that quercetin may play an important role in EV71-induced critically severe and fatal cases.

## Conclusions

Quercetin can effectively prevent EV71-induced cell injury with low toxicity to host cells. Based on our findings, quercetin may act in more than one way to deter viral infection, exhibiting some preventive and a powerful therapeutic effect against EV71. Further, this study demonstrates that quercetin potently inhibits EV71 3C^pro^ activity, thereby blocking EV71 replication.
